# Evaluation of cumulative dose for cone‐beam computed tomography (CBCT) scans within phantoms made from different compositions using Monte Carlo simulations

**DOI:** 10.1120/jacmp.v16i6.5793

**Published:** 2015-11-08

**Authors:** Abdullah Abuhaimed, Colin J. Martin, Marimuthu Sankaralingam, Kurian Oomen, David J. Gentle

**Affiliations:** ^1^ Radiotherapy Physics Department of Clinical Physics and Bioengineering, Beatson West of Scotland Cancer Center Glasgow UK; ^2^ Department of Clinical Physics University of Glasgow Glasgow UK; ^3^ Department of Applied Physics King Abdulaziz City for Science and Technology Riyadh Saudi Arabia; ^4^ Health Physics Department of Clinical Physics and Bioengineering, Gartnavel Royal Hospital Glasgow UK

**Keywords:** cumulative dose, CBCT, Monte Carlo simulation, efficiency, conversion factors, AAPM TG‐111

## Abstract

Measurement of cumulative dose f(0,150) with a small ionization chamber within standard polymethyl methacrylate (PMMA) CT head and body phantoms, 150 mm in length, is a possible practical method for cone‐beam computed tomography (CBCT) dosimetry. This differs from evaluating cumulative dose under scatter equilibrium conditions within an infinitely long phantom f(0,∞), which is proposed by AAPM TG‐111 for CBCT dosimetry. The aim of this study was to investigate the feasibility of using f(0,150) to estimate values for f(0,∞) in long head and body phantoms made of PMMA, polyethylene (PE), and water, using beam qualities for tube potentials of 80−140 kV. The study also investigated the possibility of using 150 mm PE phantoms for assessment of f(0,∞) within long PE phantoms, the ICRU/AAPM phantom. The influence of scan parameters, composition, and length of the phantoms was investigated. The capability of f(0,150) to assess f(0,∞) has been defined as the efficiency and assessed in terms of the ratios ϵ(f(0,150)/f(0,∞)). The efficiencies were calculated using Monte Carlo simulations for an On‐Board Imager (OBI) system mounted on a TrueBeam linear accelerator. Head and body scanning protocols with beams of width 40−500 mm were used. Efficiencies ϵ(PMMA/PMMA) and ϵ(PE/PE) as a function of beam width exhibited three separate regions. For beam widths <150 mm, ϵ(PMMA/PMMA) and ϵ(PE/PE) values were greater than 90% for the head and body phantoms. The efficiency values then fell rapidly with increasing beam width before levelling off at 74% for ϵ(PMMA/PMMA) and 69% for ϵ(PE/PE) for a 500 mm beam width. The quantities ϵ(PMMA/PE) and ϵ(PMMA/Water) varied with beam width in a different manner. Values at the centers of the phantoms for narrow beams were lower and increased to a steady state for ∼100−150 mm wide beams, before declining with increasing the beam width, whereas values at the peripheries decreased steadily with beam width. Results for ϵ(PMMA/PMMA) were virtually independent of tube potential, but there was more variation for ϵ(PMMA/PE) and ϵ(PMMA/Water). f(0,150) underestimated f(0,∞) for beam widths used for CBCT scans, thus it is necessary to use long phantoms, or apply conversion factors ($C_{f}s$) to measurements with standard PMMA CT phantoms. The efficiency values have been used to derive ($C_{f}s$) to allow evaluation of f(0,∞) from measurements of f(0,150). The ($C_{f}s$) only showed a weak dependence on scan parameters and scanner type, and so may be suitable for general application.

PACS number: 87.55.K‐, 87.57.Q‐, 87.57.uq.

## I. INTRODUCTION

Cone‐beam computed tomography (CBCT) scans are generally performed using a flat‐panel detector system. They enable sectional images to be obtained on equipment such as C‐arm units or kV imaging systems mounted on linear accelerators. The CT dose index (CTDI) has been the main dose metric applied to CT dosimetry. This requires normalization of an integrated dose profile for a single axial rotation with respect to the nominal beam width.^(1)^ This requires the dose profile across the beam to be integrated over an arbitrary length of 100 mm,^(2)^ and is measured using a 100 mm long pencil ionization chamber. Other dosimetric quantities designed to relate more closely to patient doses are based on ${\text{CTDI}}_{100}$ measurements performed using the pencil chambers placed inside cylindrical phantoms made of polymethyl methacrylate (PMMA). The standard PMMA phantoms, which are widely available, are 150 mm in length, and 160 mm and 320 mm in diameter, with the small phantom representing an adult head or a pediatric body, and the larger phantom an adult body. Measurements are made with the phantoms placed at the middle of the scan axis to measure ${\text{CTDI}}_{100}$ at the center and periphery, and the weighted CTDI (${\text{CTDI}}_{w}$) is calculated, based on a 2:1 weighting of the peripheral and central measurements.

As ${\text{CTDI}}_{100}$ is evaluated with a chamber and phantoms of arbitrary lengths, the beam width used for a CT scan plays a major role in determining the ${\text{CTDI}}_{100}$ value. The ${\text{CTDI}}_{100}$ at the center of the head and body phantoms is only able to detect ∼75% and ∼60%, respectively, of the total dose that would be delivered to a longer phantom, defined as ${\text{CTDI}}_{\infty }$, for narrow beams, because radiation scattered to the side is not detected by the 100 mm chamber.^3^, ^4^, ^5^, ^6^, ^7^, ^8^ The proportion recorded decreases as beam width increases, approaching ∼25% for wider beams.^9^, ^10^, ^11^ The underestimation of ${\text{CTDI}}_{100}$ with wider beams, such as those used for cone‐beam CT (CBCT) scans, is substantial as the beam width becomes larger than the length of the chamber (>100 mm), so that part of the primary beam, as well as a large portion of the scattered radiation, is not detected. The underestimation becomes more significant when beams of width >150 mm are employed, as the beam is then wider than the standard PMMA phantoms length.

The issue surrounding the use of the ${\text{CTDI}}_{100}$ for CBCT dosimetry has become more urgent with the growth in CBCT use, and it has received attention from several international organizations and the research community. Different practical solutions have been proposed to overcome the ${\text{CTDI}}_{100}$ limitation. The American Association of Physicists in Medicine (AAPM) TG–111 have proposed a method^(12)^ based on measurement of the cumulative dose for a CT or CBCT scan at the middle of a cylindrical phantom made of PMMA, polyethylene (PE) or water, of a sufficient length (≥450 mm) to capture most of the radiation.^(12)^ Furthermore, the AAPM TG–200^(13)^ and the International Commission on Radiation Units and Measurements (ICRU) Report–87^(14)^ have recommended a cylindrical phantom, named the ICRU/AAPM phantom, for measurement of cumulative dose for body scans of adult patients. This phantom is made of PE, 300 mm in diameter and 600 mm in length, and is manufactured with central and peripheral axes similar to those of the standard PMMA phantoms. The long phantoms are large and heavy, and are less practical for regular dose measurements in the clinic environment. However, if correction factors could be derived to allow cumulative dose in longer phantoms to be derived from measurements within the standard PMMA phantoms, this has the potential to provide a more practical method for CBCT dosimetry.^14^, ^15^, ^16^

The present study was conducted using Monte Carlo (MC) simulations, in order to allow a wide range of beam conditions and phantom sizes and compositions to be studied. It aimed to investigate the possibility of using a small chamber within the standard PMMA phantoms to evaluate cumulative doses for CBCT scans measured under equilibrium scatter conditions within long phantoms,^(12)^ through the application of correction factors. The influence of beam width 40−500 mm and beam quality for tube potentials of 80−140 kV on the relationship between the cumulative dose values for short and long phantoms has been studied. As the density of PE is lower than that for PMMA, and it is widely available, the present study also investigated the suitability of using 150 mm PE phantoms for assessment of cumulative dose within the long PE phantoms. The relationships between the short and long phantoms, studied using the different scans parameters, were fitted to polynomial equations, from which conversion factors were derived to allow evaluation of the cumulative dose within a long phantom from single measurements made within the standard PMMA phantoms.

## II. MATERIALS AND METHODS

### A. Cone‐beam CT system

A kV imaging system utilized to acquire CBCT scans for patients positioning and adaptive radiotherapy in the image‐guided radiation therapy (IGRT) was employed for this study. The kV system is known as On‐Board Imager (OBI), and is incorporated into a Varian TrueBeam linear accelerator (Varian Medical systems, Palo Alto, CA). The OBI system has been described in detail in previous studies.^11^, ^15^ Two scanning protocols (head and body) with different parameters were used. Both were acquired for full 360° rotations, the main differences between the protocols being in the bowtie filter type and the diameter of the scan (i.e., field of view). In the clinic, the full‐fan mode with a full bowtie filter and a scan diameter of 264 mm is employed to scan smaller objects, while the half‐fan mode with a half bowtie filter and a scan diameter of 478 mm allows a scan to be acquired for larger objects. Therefore, the full‐ and half‐fan modes were used for the head and body protocols, respectively. The nominal beam widths used for the scans ranged from 40 mm to 500 mm, with an increment of 20 mm, along the rotation axis (z‐axis). Four different beam qualities at tube potentials of 80 kV, 100 kV, 120/125 kV, and 140 kV were used. 125 kV was chosen for body scanning as this is used in clinic protocols.

### B. Cumulative dose for CBCT scans

The method proposed by AAPM requires measuring the cumulative dose for CBCT scans as a point dose f(0) at the peak of a dose profile resulting from a single CBCT rotation. For scans obtained with multiple rotations, the cumulative dose is assessed by multiplying f(0) by the number of rotations (N) involved in the scan, Nf(0). In the present study, only one rotation was used for all the scans, thus N=1. f(0) is measured using a small chamber such as a $0.6\;{\text{cm}}^{3}$ Farmer‐type ionization chamber at the middle of a phantom, that is sufficiently long to create the scatter equilibrium condition under which the cumulative dose is measured. Like ${\text{CTDI}}_{100}$ measurements, f(0) is measured at the center of the phantom $f({0)}_{c}$, and four peripheral positions $f({0)}_{p}$ located 1 cm below the phantom surface, where $f({0)}_{p}$ is the average for the four peripheral measurements. In this study, f(0,∞) was used instead of f(0) to distinguish between the cumulative dose values measured in 150 mm long phantoms f(0,150) and those in infinitely long phantoms f(0,∞).

### C. The efficiency of f(0,150)

In order to investigate the capability of measurements performed with a small chamber within standard PMMA phantoms to evaluate cumulative doses measured in infinitely long phantoms, f(0,150) was normalized with respect to f(0,∞). f(0,∞) values were calculated in phantoms made from PMMA, PE, and water, and the results are expressed in terms of the efficiency of the standard PMMA phantoms in recording f(0,∞) as:^(17)^

(1) $$\varepsilon {\left(PMMA/m\right)}_{x,kV}=\frac{f{\left(0,150\right)}_{x,PMMA}}{f{\left(0,\infty \right)}_{x,m}}$$

where $f(0,{150)}_{x,PMMA}$ and $f(0,{\infty )}_{x,m}$ were calculated with the same beam widths, scan parameters, and positions within the phantoms; *m* relates to the composition of the infinitely long phantom (PMMA, PE or water), *x* represents the position of the measurements within the phantom (center or periphery), and *kV* is the tube potential used for the measurements.

The influence of the tube potential in the range 80−140 kV on $\epsilon (\text{PMMA}/{m)}_{x,\text{kV}}$ values was investigated by normalizing ε(PMMA/m)_x,80kV_, $\epsilon (\text{PMMA}/{m)}_{x,100\text{kV}}$, and $\epsilon (\text{PMMA}/{m)}_{x,140\text{kV}}$, values with respect to those of $\epsilon (\text{PMMA}/{m)}_{x,120\text{kV}}$ for the head phantom and $\epsilon (\text{PMMA}/{m)}_{x,125\text{kV}}$ for the body phantom as follows:

(2) $$Head=\frac{\varepsilon {\left(PMMA/m\right)}_{x,kV}}{\varepsilon {\left(PMMA/m\right)}_{x,120kV}},Body=\frac{\varepsilon {\left(PMMA/m\right)}_{x,kV}}{\varepsilon {\left(PMMA/m\right)}_{x,125kV}}$$

The suitability of a 150 mm long PE phantom $\epsilon (\text{PE}/{\text{PE})}_{x,\text{kV}}$ for assessment of cumulative doses in long PE phantom was investigated in a similar manner and results presented in the form:

(3) $$\varepsilon {\left(PE/PE\right)}_{x,kV}=\frac{f{\left(0,150\right)}_{x,PE}}{f{\left(0,\infty \right)}_{x,PE}}$$

### D. Conversion factors for f(0,∞)

From the efficiency values obtained in Materials & Methods section C, conversion factors ($C_{f}s$) for beams of width 40−500 mm and tube potentials of 80−140 kV were derived, which allows the evaluation of f(0,∞) from single measurements made within standard PMMA phantoms $f(0,{150)}_{x,\text{PMMA}}$. $C_{f}s$ were derived as:

(4) $$C_{f}{\left(PMM/m\right)}_{x,kV}=\frac{1}{\varepsilon {\left(PMMA/m\right)}_{x,kV}}=\frac{f{\left(0,\infty \right)}_{x,m}}{f{\left(0,150\right)}_{x,PMMA}}$$

Once $C_{f}(\text{PMMA}/{m)}_{x,\text{kV}}$ is known, f(0,∞) for a specific beam width and at a given tube potential can then be assessed as:

(5) $$f{\left(0,\infty \right)}_{x,m}=C_{f}{\left(PMMA/m\right)}_{x,kV}\times f{\left(0,150\right)}_{x,PMMA}$$

### E. Monte Carlo simulations

The Monte Carlo (MC) technique enables a wide range of dosimetric studies that would be more difficult and time‐consuming to investigate experimentally. Values for $f(0,{150)}_{x,\text{PMMA}}$, $f(0,{150)}_{x,\text{PE}}$, and $f(0,{\infty )}_{x,m}$ were assessed using MC EGSnrc–based user codes (V4‐r2‐4‐0, National Research Council of Canada, Ottawa, Canada)^18^, ^19^, ^20^ namely BEAMnrc^21^, ^22^ and DOSXYZnrc.^(23)^ Although the MC EGSnrc system is communally utilized to simulate MV applications such as linear accelerators, kV applications can also be simulated.^24^, ^25^, ^26^ Many studies have shown that BEAMnrc and DOSXYZnrc codes simulated and calculated absorbed doses resulting from CBCT scans employed in IGRT procedures with a high degree of accuracy.^27^, ^28^, ^29^ The OBI system, described in Materials & Methods section A, was designed using BEAMnrc, and the absorbed dose within head and body phantoms of different compositions was calculated with DOSXYZnrc. Experimental measurements validating the MC model developed in BEAMnrc and the absorbed dose values calculated with DOSXYZnrc were reported in previous studies.^11^, ^15^ The comparison showed the experimental measurements and MC results to be in good agreement. The additional MC simulations in the present study were accomplished in two stages, described in the following subsections:

#### E.1 BEAMnrc simulations

In this stage, BEAMnrc was used to simulate the kV source using the head and body protocols. Monoenergetic electron beams with a diameter of 1 mm and energies of 80−140 keV were simulated using ISOURCE=10(parallel circular beam incident from side) of the code to accelerate electrons towards the anode of the OBI system from the side at an angle of 14°. $0.85-1\times 10^{9}$ histories were run for 192 simulations, 96 for each protocol, at different tube potentials and fields of sizes 264 mm×40−500 mm for the head protocol and478 mm×40−500 mm for the body protocol, where 264 and 478 mm were diameters of the scans and 40−500 mm were the scan beam widths along the rotation axis (z‐axis). All the MC parameters used for BEAMnrc simulations, such as the low‐energy thresholds for creation of secondary electrons (AE) and photons (AP), the cutoff energies for transport of the electrons (ECUT) and photons (PCUT), the directional bremsstrahlung splitting (DBS) technique, and the cross‐sectional data, were similar to those used in previous studies.^11^, ^15^ The output file for each simulation, which is known as the phase space (PHSP) file, was recorded at a source‐surface‐distance (SSD) of 75 cm. A library containing the resulting 192 PHSP files was subsequently created and these were used as sources for simulations carried out with DOSXYZnrc in the second stage.

#### E.2 DOSXYZnrc simulations

In the second stage, the library of PHSP files was employed to run 816 simulations, 408 for each protocol, using ISOURCE=8(phase‐space source incident from multiple directions) to calculate $f(0,{150)}_{x,\text{PMMA}}$, $f(0,{150)}_{x,\text{PE}}$, and $f(0,{\infty )}_{x,m}$ values within 10 different phantoms. Five phantoms — (1) short and (2) infinitely long $\text{PMMA}(C_{5}O_{2}H_{8},\rho =1.19g/{\text{cm}}^{3})$ phantoms, (3) short and (4) infinitely long PE phantoms ($C_{2}H_{4},\rho =0.97g/{\text{cm}}^{3}$), and (5) infinitely long water phantoms ($H_{2}O,\rho =1.0g/{\text{cm}}^{3}$) — were designed in DOSXYZnrc for each protocol. Diameters and lengths of the phantoms used in this study are shown in Table 1. Diameters of the standard and infinitely long PMMA phantoms were similar to those used in the clinic. The head PE phantom was selected as it is equivalent to the standard head PMMA phantom,^(30)^ and the body PE phantom was designed to emulate the ICRU/AAPM phantom.^(14)^ Diameters of the head and body water phantoms were as recommended by AAPM TG–111.^(12)^ The short phantoms were designed with a length of 150 mm, while the phantoms simulating the infinite length were 600 mm in length as this has been found to be sufficient to create the scatter equilibrium condition required for the cumulative dose measurements.^12^, ^14^

**Table 1:** Diameters and lengths of the phantoms used in this study

*Phantom Composition*	*Head Diameter (mm)*	*Body Diameter (mm)*	*Phantom Length (mm)*
PMMA	160	320	150
			600
PE	160	300	150
			600
Water	200	300	600

$5-6\times 10^{8}$ histories with a photon splitting number of 50 were run for each simulation to obtain a statistical uncertainty of less than 1%. All the MC parameters used for DOSXYZnrc simulations such as AE, ECUT, AP, PCUT, the cross‐sectional data, voxels size, and the use of HOWFARLESS transport algorithm were similar to those used in previous studies.^11^, ^15^ Output files of the simulations were analyzed using a MATLAB–based code (MathWorks, Natick, MA) developed in house. PEGS4 code was used to generate properties of all the materials used in the simulations in the two stages. All the simulations carried out in this study were run in the Scottish Grid Service (ScotGrid) at the University of Glasgow.

### F. Experimental measurements for $f(0,{150)}_{x,\text{PMMA}}$ and $f(0,{\infty )}_{x,\text{PMMA}}$

In the clinic, CBCT scans are acquired using two different acquisition modes: a partial scan with a 200° rotation or a full scan with a 360° rotation. In order to examine the sensitivity of the conversion factors derived in Materials & Methods section D to the scan parameters such as the type of bowtie filter, the scan diameter, and the scan acquisition mode, experimental measurements for $f(0,{150)}_{x,\text{PMMA}}$ and $f(0,{\infty )}_{x,\text{PMMA}}$ using head and body phantoms were performed using four different scanning protocols as listed in Table 2. $f(0,{150)}_{x,\text{PMMA}}$ was measured within the standard PMMA phantoms, while $f(0,{\infty )}_{x,\text{PMMA}}$ was measured within 450 mm long PMMA phantoms formed by combining three standard PMMA phantoms end to end.^(12)^ The phantoms were set up with the central axis at the isocenter with a source‐to‐isocenter distance (SID) of 100 cm. $f(0,{150)}_{x,\text{PMMA}}$ and $f(0,{\infty )}_{x,\text{PMMA}}$ were measured at the middle of the central and four peripheral axes of the phantoms using a $0.6\;{\text{cm}}^{3}$ Farmer–type ionization chamber (10X5‐0.6CT, Radcal Corporation, Monrovia, CA) with a calibration traceable to a standard dosimetry laboratory for beam qualities in the kV range.

**Table 2:** Scanning parameters employed for experimental measurements for $f(0,{150)}_{x,\text{PMMA}}$ and $f(0,{\infty )}_{\text{xPMMA}}$ values using the standard and long head and body PMMA phantoms

	*Full Head*	*Partial Head*		*Full Body*	*Partial Body*
Tube potential (kV)	100	100		125	125
Scan trajectory	360°	200°		360°	200°
Bowtie filter	Full	Full		Half	Full
Scan diameter (mm)	264	264		478	264
Scan beam width (mm)			198		

## III. RESULTS & DISCUSSION

### A. The efficiency values for $\epsilon (\text{PMMA}/{\text{PMMA})}_{x,\text{kV}}$

**Figure 1:**
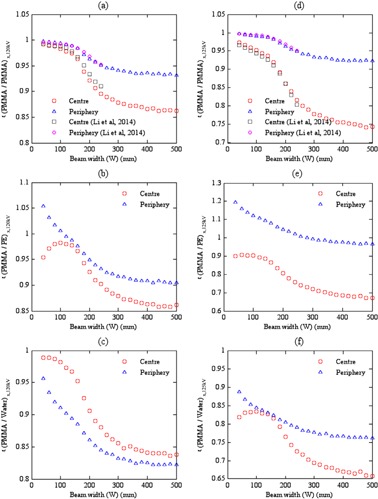
The efficiency values calculated as in Eq. (1) for (a) $\epsilon (\text{PMMA}/{\text{PMMA})}_{x,120\text{kV}}$, (b) $\epsilon (\text{PMMA}/{\text{PE})}_{x,120\text{kV}}$, and (c) $\epsilon (\text{PMMA}/{\text{Water})}_{x,120\text{kV}}$ at the center and periphery of the head phantoms at 120 kV, and (d) to (f) for the body phantoms at 125 kV. $\epsilon (\text{PMMA}/{\text{PMMA})}_{x,\text{kV}}$ values for the head and body phantoms ((a) and (d)) were compared to those of Li et al.^(17)^ obtained within PMMA phantoms using a Somatom Definition dual source CT scanner at 120 kV and beams of width 30−250 mm.

Figure 1 shows the efficiency values $\epsilon (\text{PMMA}/{m)}_{x,\text{kV}}$, calculated using Eq. (1), that compare measurements in the standard PMMA phantom with the infinitely long PMMA, PE, and water head phantoms at 120 kV and body phantoms at 125 kV. $\epsilon (\text{PMMA}/{\text{PMMA})}_{x,\text{kV}}$ values at the central and peripheral axes declined as the beam width increased (Figs. 1(a) and (d)). The central $\epsilon (\text{PMMA}/{\text{PMMA})}_{c,\text{kV}}$ values for the head phantom were greater than those for the body, and the peripheral $\epsilon (\text{PMMA}/{\text{PMMA})}_{p,\text{kV}}$ values were almost identical for the head and body phantoms, but higher than those at the center. The differences at the centers of the phantoms arose from the differences in phantom diameters. There was less attenuation of the primary beam in the head phantom, so the scatter formed a proportionately smaller component of the measurement at the center.

In addition, Dixon and Boone^(31)^ showed that the scatter tails in the dose profile from a beam of width 28 mm at the center of a PMMA body phantom extended to ±200 mm (i.e., 400 mm) along the rotation axis (z‐axis). Therefore, the scatter tails extend beyond the length of the standard phantoms; thus $f(0,{150)}_{x,\text{PMMA}}$ will not record the entire absorbed dose that would be deposited in a longer phantom even for a narrow beam (Figs. 1(a) and (d)). The extents of the scatter tails, although related to the nominal beam width, are influenced by factors such as tube potential, phantom composition and diameter, the position within the phantom at which the measurements are made, and the use of a bowtie filter.^14^, ^32^, ^33^ This, therefore, affects the scatter to primary ratio (SPR) values at the center and periphery of the head and body phantoms.^(32)^ For example, the SPR for a narrow beam at the center of the standard body PMMA phantom was higher by a factor of ∼7 than that at the periphery, and greater by a factor of ∼3 than that at the center of the standard PMMA head phantom.^(34)^ This in turn was larger by a factor ∼2 than those at the periphery of the head phantom. The variations in SPR values, and the inability of the standard phantoms to detect the whole scatter tails, determine the variations in $\epsilon (\text{PMMA}/{\text{PMMA})}_{x,\text{kV}}$ values between the central and peripheral axes and between the phantoms.

The relationship between $\epsilon (\text{PMMA}/{\text{PMMA})}_{x,\text{kV}}$ and beam width can be divided into three regions (Figs. 1(a) and (d)). The first region extended from narrow beam widths up to ∼150 mm. In this region, the whole primary beam was within the phantom length. Therefore, both $f(0,{150)}_{x,\text{PMMA}}$ and $f(0,{\infty )}_{x,\text{PMMA}}$ values increased with beam width, although the contribution from scattered radiation to $f(0,{\infty )}_{x,\text{PMMA}}$ increased more rapidly than that to $f(0,{150)}_{x,\text{PMMA}}$. The second region began at beam widths >150 mm, when the beam extended beyond the length of the standard PMMA phantoms, so that a part of the primary beam did not contribute to the scattered radiation. As a result, $f(0,{150)}_{x,\text{PMMA}}$ values were virtually constant, as further increases in beam width made a negligible contribution to measurements at z=0. In contrast, $f(0,{\infty )}_{x,\text{PMMA}}$ continued to rise, and so $\epsilon (\text{PMMA}/{\text{PMMA})}_{x,\text{kV}}$ declined at both the center and periphery of the phantoms. The third region began when further increases in beam width led to minimal contributions to $f(0,{\infty )}_{x,\text{PMMA}}$ values. This occurred when the beam width approached the equilibrium beam width ($a_{\text{eq}}$) at which further increases in beam width made a negligible contribution to $f(0,{\infty )}_{x,\text{PMMA}}$ as the scattered radiation did not reach the middle of the phantom (z=0).^12^, ^35^ The equilibrium beam width depends on the phantom diameter and the position within the phantom.^12^, ^14^ Since the increase in $f(0,{\infty )}_{x,\text{PMMA}}$ in this region was much lower compared to the second region, the decline in $\epsilon (\text{PMMA}/{\text{PMMA})}_{x,\text{kV}}$ was less and values became virtually constant.

Li et al.^(17)^ studied $\epsilon (\text{PMMA}/{\text{PMMA})}_{x,\text{kV}}$ values for a Somatom Definition dual source CT scanner (Siemens Healthcare, Malvern, PA) using MC simulations. The efficiency values were investigated for standard and 900 mm long head and body PMMA phantoms of the same diameters used in this study, for a tube potential of 120 kV, a full 360° rotation scan, head and body bowtie filters, and beams ranging in width from 30 mm to 250 mm. As shown in Figs. 1(a) and (d), $\epsilon (\text{PMMA}/{\text{PMMA})}_{x,\text{kV}}$ values from this study are in good agreement with those of Li et al.^(17)^ within ±1.53% and ±0.56% at the center and periphery of the head phantom with variations of the mean of 0.72% and 0.10%, respectively, and within ±1.56% and ±0.55% for the body phantom with variations of the mean of 0.88% and 0.28%, respectively. Although the kV systems (CT and CBCT scanners) and the length of the infinitely long phantoms were different in the two studies, the differences are minor.

### B. The efficiency values for $\epsilon (\text{PMMA}/{\text{PE})}_{x,\text{kV}}$ and $\epsilon (\text{PMMA}/{\text{Water})}_{x,\text{kV}}$

Figures 1(b) and (e) and Figs. 1(c) and (f) show the efficiency values for $\epsilon (\text{PMMA}/{\text{PE})}_{x,\text{kV}}$ and $\epsilon (\text{PMMA}/{\text{Water})}_{x,\text{kV}}$, respectively. Although there are similarities in form with results comparing measurements in standard PMMA phantoms, there are substantial differences. It should be noted that $\epsilon (\text{PMMA}/{\text{PMMA})}_{x,\text{kV}}$ results were for phantoms of similar diameter and composition, and so were only influenced by differences in the lengths of the phantoms. However, the $\epsilon (\text{PMMA}/{\text{PE})}_{x,\text{kV}}$ and $\epsilon (\text{PMMA}/{\text{Water})}_{x,\text{kV}}$ values are from comparisons of phantoms with different diameters, compositions, and lengths. Differences in diameter will affect attenuation of the transmitted beam reaching the center, while differences in phantom material will alter the mass attenuation coefficients and mass energy‐absorption coefficients, and hence both the attenuation and the extent of the scatter tails.^30^, ^32^

PMMA has a higher attenuation than both PE and water, and the PMMA body phantom has a larger diameter (Table 1). Differences in the primary radiation transmitted to the centers of the phantoms are apparent in the lower ratios for narrow beams (Figs. 1(b)–(c) and (e)–(f)). The scatter component in measurements at the centers increased with beam width, thus the influence of attenuation of the primary beam on dose level at the center then declined, and the efficiency values increased accordingly up to beam widths of the order of 100 mm. Thereafter, the efficiency versus beam‐width relationships became similar to that from comparisons of PMMA phantoms (Figs. 1(a) and (d)), and followed similar relationship in the second and third regions. The peripheral measurements for narrow beams were affected by the different intensities incident on the phantom surfaces, resulting from the different compositions, and hence the scattering properties (Table 1). The ratios between PMMA and other materials declined with beam width more than the PMMA ratios. The lower peripheral than center head phantom ratio for the water head phantom was a result of the large difference in PMMA and water phantom diameters, as well as the composition (Fig. 1(c) and Table 1).

### C. The efficiency values for $\epsilon (\text{PE}/{\text{PE})}_{x,\text{kV}}$

**Figure 2:**
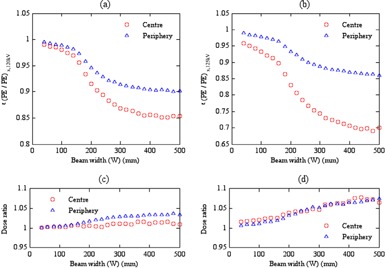
The efficiency values for $\epsilon (\text{PE}/{\text{PE})}_{x,\text{kV}}$ calculated as in Eq. (3) within (a) head phantoms at 120 kV and (b) body phantoms at 125 kV. The dose ratios ((c) and (d)) for $\epsilon (\text{PMMA}/{\text{PMMA})}_{x,\text{kV}}$ values normalised with respect to $\epsilon (\text{PE}/{\text{PE})}_{x,\text{kV}}$ values at the same tube potential and position within (c) head and (d) body phantoms.

Comparisons were made between shorter and longer PE phantoms, as PE might present an alternative for standard hospital dosimetry phantoms. Figure 2 ((a) and (b)) show $\epsilon (\text{PE}/{\text{PE})}_{x,\text{kV}}$ values at the center and periphery of the head and body phantoms as a function of beam width, calculated using Eq. (3). Because $\epsilon (\text{PE}/{\text{PE})}_{x,\text{kV}}$ values were only affected by the difference in phantom lengths, the trends for $\epsilon (\text{PE}/{\text{PE})}_{x,\text{kV}}$ were similar to those for $\epsilon (\text{PMMA}/{\text{PMMA})}_{x,\text{kV}}$ (Figs. 1(a) and (d)). $\epsilon (\text{PE}/{\text{PE})}_{x,\text{kV}}$ values also exhibited the three regions observed for $\epsilon (\text{PMMA}/{\text{PMMA})}_{x,\text{kV}}$. However, $\epsilon (\text{PE}/{\text{PE})}_{x,\text{kV}}$ values within the head at 120 kV and body at 125 kV were lower than those for $\epsilon (\text{PMMA}/{\text{PMMA})}_{x,\text{kV}}$ at the same tube potentials by up to 1% and 3% at the center and periphery of the head phantom, respectively, and 6% and 7% within the body phantom, respectively (Figs. 2(c) – (d)). The variations between $\epsilon (\text{PE}/{\text{PE})}_{x,\text{kV}}$ and $\epsilon (\text{PMMA}/{\text{PMMA})}_{x,\text{kV}}$ values were found to increase with beam width beyond 150 mm (i.e., the length of the short phantoms). This is caused by the increase of buildup of scattered radiation within the infinitely long PE phantom with increasing beam width due to the lower density compared to PMMA.^(14)^ PE phantoms have broader dose spread functions (i.e., longer scatter tails^(32)^) so that the decline in $\epsilon (\text{PE}/{\text{PE})}_{x,\text{kV}}$ with beam width is greater than for $\epsilon (\text{PMMA}/{\text{PMMA})}_{x,\text{kV}}$. This means that the dose underestimation with standard PMMA phantoms will be less than with short PE phantoms. Thus, the current PMMA phantoms provide a better option for dosimetry $f(0,{\infty )}_{x,\text{PMMA}}$.

### D. The influence of the tube potential on the efficiency values

**Figure 3:**
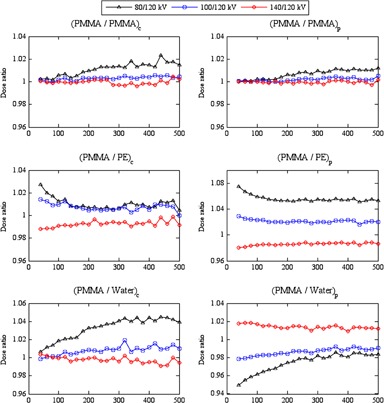
The influence of tube potential on the efficiency values $\epsilon (\text{PMMA}/{\text{PMMA})}_{x,\text{kV}}$, $\epsilon (\text{PMMA}/{\text{PE})}_{x,\text{kV}}$, and $\epsilon (\text{PMMA}/{\text{Water})}_{x,\text{kV}}$ at the center, c, and periphery, p, of the head phantoms. The efficiency values calculated at 80, 100, and 140 kV were normalized with respect to those for 120 kV.

**Figure 4:**
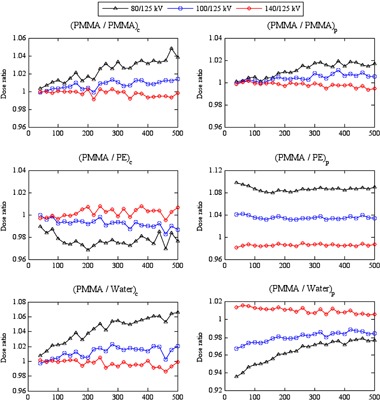
The influence of tube potential on the efficiency values $\epsilon (\text{PMMA}/{\text{PMMA})}_{x,\text{kV}}$, $\epsilon (\text{PMMA}/{\text{PE})}_{x,\text{kV}}$, and $\epsilon (\text{PMMA}/{\text{Water})}_{x,\text{kV}}$ at the center, c, and periphery, p, of the body phantoms. The efficiency values calculated at 80, 100, and 140 kV were normalized with respect to those for 125 kV.

Figures 3 and 4 show the influence of tube potential on the efficiency values $\epsilon (\text{PMMA}/{m)}_{x,\text{kV}}$ at the center and periphery of the head and body phantoms, respectively. Differences in efficiency with tube potential were less for $\epsilon (\text{PMMA}/{\text{PMMA})}_{x,\text{kV}}$, where the composition is the same, but larger for phantoms of different compositions $\epsilon (\text{PMMA}/{\text{PE})}_{x,\text{kV}}$ and $\epsilon (\text{PMMA}/{\text{Water})}_{x,\text{kV}}$. The influence of tube potential was greater within the body phantoms (Fig. 4) than the head phantoms (Fig. 3). These variations resulted from the difference in diameters and compositions of the head and body phantoms and in the scatter tails with tube potentials 80−140 kV.^(32)^

For $\epsilon (\text{PMMA}/{\text{PMMA})}_{x,\text{kV}}$, the efficiency values for 80 kV were larger by up to 4.8% than for other tube potentials, but the differences between the values for 100−140 kV were within ±1%, which is in agreement with results of Li et al.^(17)^ The differences in efficiency values $\epsilon (\text{PMMA}/{\text{PMMA})}_{x,\text{kV}}$ with tube potential were similar at the centers and peripheries of the head and body phantoms, and increased with beam width, with the variations for the body phantom being slightly larger. The increase of the differences with the beam width resulted from the buildup of the scattered radiation. Differences in efficiency ratios for phantoms of different dimensions and compositions were larger, being up to ±10.5% for $\epsilon (\text{PMMA}/{\text{PE})}_{x,\text{kV}}$ and ±6.9% for $\epsilon (\text{PMMA}/{\text{Water})}_{x,\text{kV}}$ within the head and body phantoms. These larger variations were caused by the differences between the properties of the phantoms. For the peripheral measurements, values for $\epsilon (\text{PMMA}/{\text{PE})}_{p,\text{kV}}$ at 80 kV were larger for both head and body phantoms, while values for $\epsilon (\text{PMMA}/{\text{Water})}_{p,\text{kV}}$ at 140 kV were larger. Ratios at the centers of the body phantoms $\epsilon (\text{PMMA}/{\text{PE})}_{c,\text{kV}}$ and $\epsilon (\text{PMMA}/{\text{Water})}_{c,\text{kV}}$ were the reverse of this, linked to the higher photoelectric component in the attenuation of 80 kV X‐rays in water than in PMMA, and in PMMA than in PE.^36^, ^37^ However, the trends in $\epsilon (\text{PMMA}/{\text{PE})}_{c,\text{kV}}$ and $\epsilon (\text{PMMA}/{\text{Water})}_{c,\text{kV}}$ for the head phantom did not show the same patterns, but the variations in dose within these phantoms are much smaller than the body phantoms.

### E. Conversion factors for $f(0,{\infty )}_{x,m}$

From the efficiency values calculated in the Results & Discussion sections A and B and the investigation of the influence of tube potential shown in section D, conversion factors have been derived using Eq. (4) to enable the evaluation of $f(0,{\infty )}_{\text{xm}}$ based on measurements made in the standard PMMA phantoms. Conversion factors $C_{f}(\text{PMMA}/{\text{PMMA})}_{\text{kV}}$, $C_{f}(\text{PMMA}/{\text{PE})}_{x,\text{kV}}$, and $C_{f}(\text{PMMA}/{\text{Water})}_{x,\text{kV}}$ have been derived for the center and periphery of each phantom for tube potentials from 80 kV to 140 kV. The conversion factor data were fitted to sixth‐degree polynomial equations with $R^{2}>0.99$ for beams of width 40−500 mm. The coefficients of the fitted equations are given in Appendix A, Tables A.1 to A.3.

The conversion factors are only suitable for application to cumulative dose measured $f(0,{150)}_{x,\text{PMMA}}$ with a small chamber such as 20 mm. The use of a 100 mm pencil ionization chamber would lead to large variations especially for narrow beam widths.^(15)^ For example, $f(0,{150)}_{x,\text{PMMA}}$ values measured with a small chamber for a beam of width 40 mm at 125 kV at the center and periphery of the PMMA body phantom are larger by factors of 1.6 and 2.3, respectively, than those measured with the 100 mm chamber. However, $f(0,{150)}_{x,\text{PMMA}}$ can be evaluated from measurements made with the 100 mm pencil ionization chamber by using a function called G_x_(W)_100_.^(16)^

### F. Sensitivity of conversion factors for the kV system and scan parameters

Table 3 compares experimental measurements of $f(0,{150)}_{x,\text{PMMA}}$ and $f(0,{\infty )}_{x,\text{PMMA}}$ using the scanning protocols listed in Table 2, and $f(0,{\infty )}_{x,m}$ values evaluated by using the conversion factors derived from MC calculations provided in Tables A.1 to A.3 of the Appendix. The differences between $f(0,{\infty )}_{x,\text{PMMA}}$ values measured experimentally and those evaluated by application of the conversion factors using Eq. (5) were within ±2.9% and ±2.5% for the head and body phantoms, respectively (Table 3). The larger differences occurred at the centers of the phantoms, but these variations will be less in the weighted values, which average the dose over the scan plane (x and y) axes by taking one‐third of the dose at the center of the phantom and two‐thirds at the periphery. Although the MC calculations employed phantoms 600 mm in length and the experimental measurements were in 450 mm long phantoms, the differences between the experimental and calculated $f(0,{\infty )}_{x,\text{PMMA}}$ values were small. This is consistent with the recommendation of AAPM TG‐111^(12)^, where the length of the infinitely long phantom is required to be ≥450 mm to provide the scatter equilibrium condition for cumulative dose measurements. The results provide further confirmation that in practice 600 mm PMMA phantoms can be replaced by ones 450 mm in length. The use of four standard 150 mm long phantoms to create a 600 mm length would potentially leave an air gap in the middle which could produce anomalous results, whereas a 450 mm long phantom made from three standard ones avoids this issue. The differences between the values for $f(0,{\infty )}_{x,\text{PMMA}}$, $f(0,{\infty )}_{x,\text{PE}}$, and $f(0,{\infty )}_{x,\text{Water}}$ at the centers of head and body phantoms, and the peripheries of PMMA and water phantoms given in Table 3, were in agreement with results reported by Zhou and Boone^(30)^ and Li et al.^(34)^

**Table 3:** Experimental measurements for $f(0,{150)}_{x,\text{PMMA}}$ and $f(0,{\infty )}_{x,\text{PMMA}}$ performed at the center, c, and periphery, p, of standard and 450 mm long PMMA head and body phantoms using the scanning protocols given in Table 2. $f(0,{150)}_{x,\text{PMMA}}$ values were used to estimate $f(0,{\infty )}_{x,\text{PMMA}}$ within the head and body PMMA, PE, and water phantoms, using the conversion factors given in Tables A. 1 to A.3 as shown in Eq. (5). $\text{Difference}(\%)=[(f(0,{\infty )}_{\text{MC}}-f(0,{\infty )}_{\text{Exp}}/f(0,{\infty )}_{\text{Exp}})\times 100]$

	Cf−MC	$f(0,{150)}_{x,PMMA,Exp}mGy/100mAs$	$f(0,{\infty )}_{x,PMMA,Exp}mGy/100mAs$	$f(0,{\infty )}_{x,m,MC}mGy/100mAs$	*Difference (%)*	$f(0,{150)}_{x,PMMA,Exp}mGy/100mAs$	$f(0,{\infty )}_{x,PMMA,Exp}mGy/100mAs$	$f(0,{\infty )}_{x,m,MC}mGy/100mAs$	*Difference (%)*
		*Partial Head 200° scan* −100 kV				*Full Head 360° scan* −100 kV			
C‐PMMA	1.076	2.39	2.50	2.57	2.87	2.53	2.65	2.72	2.73
	1.069^a^			2.55	2.24			2.70	2.11
C‐PE	1.068			2.55				2.70	
C‐Water	1.088			2.60				2.75	
P‐PMMA	1.034	2.18	2.26	2.25	−0.24	2.27	2.34	2.35	0.33
	1.032^a^			2.25	−0.46			2.34	0.10
P‐PE	1.029			2.24				2.34	
P‐Water	1.174			2.56				2.66	
		*Partial Body 200° scan* −125 kV				*Full Body 360° scan* −125 kV			
C‐PMMA	1.153	1.59	1.83	1.83	0.20	1.37	1.62	1.58	−2.48
	1.157^a^			1.84	0.55			1.59	−2.13
C‐PE	1.235			1.96				1.69	
C‐Water	1.299			2.07				1.78	
P‐PMMA	1.036	2.30	2.35	2.38	1.42	1.91	1.98	1.98	−0.04
	1.032^a^			2.37	0.99			1.97	−0.46
P‐PE	0.953			2.19				1.82	
P‐Water	1.239			2.85				2.37	

a Conversion factor calculated from the efficiency values provided by Li et al.^(17)^ which were obtained at 120 kV using a Somatom Definition dual source CT scanner.

Table 3 also shows good agreement between experimental results with a 450 mm phantom and estimates of $f(0,{\infty )}_{x,\text{PMMA}}$ from shorter phantoms with the same conversion factors for partial (200°) scans, demonstrating that the conversion factors can also be applied to partial scans. This means that the conversion factors were relatively insensitive to the bowtie filter, the scan diameter, and the acquisition mode. The efficiency values of Li et al.^(17)^ shown in Figs. 1(a) and (d), which were calculated for a Somatom Definition dual source CT scanner at 120 kV, were also used to derive conversion factors using Eq. (4), and results using these were, again, in excellent agreement with those derived in this study. Thus, the conversion factors appear insensitive to differences in scanner type and scan parameters. These findings are consistent with previous studies,^5^, ^7^, ^8^, ^11^, ^15^, ^17^, ^38^ conducted with a wide range of CT and CBCT scanners, that show dose ratios such as f(0,150)/f(0,∞) and ${\text{CTDI}}_{100}$ / ${\text{CTDI}}_{\infty }$ to be relatively independent of scan parameters and scanner. When a dose index is normalized by another index measured with identical parameters, the influence of the scan parameters and scanner type are minimized.^14^, ^17^, ^35^ For example, dose ratios calculated with the OBI system, used in this study, were in good agreement with those measured for an earlier version of the OBI system and different CT scanners.^(15)^ This approach, therefore, provides an efficient method for developing generic coefficients, which may be suitable for a range of scanners in a manner similar to those used to estimate organ doses resulting from CT scans based on ${\text{CTDI}}_{\text{vol}}$ on the scanner console.^39^, ^40^

## IV. CONCLUSIONS

The capability of using a small chamber within the standard PMMA head and body phantoms for reporting the cumulative dose $f(0,{\infty )}_{x,m}$ within infinitely long PMMA, PE, and water phantoms has been studied using MC calculations by simulating an OBI system integrated into a TureBeam linac. The efficiency values were investigated using head and body scanning protocols over a wide range of beam widths from 40−500 mm and tube potentials of 80−140 kV. The relationships between efficiencies for shorter phantoms of the same composition $\epsilon (\text{PMMA}/{\text{PMMA})}_{x,\text{kV}}$ and $\epsilon (\text{PE}/{\text{PE})}_{x,\text{kV}}$ were similar in form, exhibiting three distinct regions, a slow decline, followed by a rapid decline, and then a leveling off. $\epsilon (\text{PMMA}/{\text{PMMA})}_{x,\text{kV}}$ values were larger than those for $\epsilon (\text{PE}/{\text{PE})}_{x,\text{kV}}$, due to differences in density. In addition to the difference in phantoms length, $\epsilon (\text{PMMA}/{\text{PE})}_{x,\text{kV}}$ and $\epsilon (\text{PMMA}/{\text{Water})}_{x,\text{kV}}$ values were also affected by differences in phantom diameters and compositions, which influenced both the attenuation and proportion of scattered photons. Tube potential had a minor influence on the efficiency values for $\epsilon (\text{PMMA}/{\text{PMMA})}_{x,\text{kV}}$, but variations were more significant for $\epsilon (\text{PMMA}/{\text{PE})}_{x,\text{kV}}$ and $\epsilon (\text{PMMA}/{\text{Water})}_{x,\text{kV}}$. The results indicated that $f(0,{150)}_{x,\text{PMMA}}$ underestimated $f(0,{\infty )}_{\text{xm}}$ values significantly for wider beams, such as those used for CBCT scans. Therefore, to measure $f(0,{\infty )}_{x,m}$, it is necessary to use long phantoms (≥450 mm), or derive conversion factors that can be applied to measurements with shorter phantoms. Therefore, based on the efficiency values calculated, conversion factors have been derived at the center and periphery of the phantoms for each tube potential to allow evaluation of $f(0,{\infty )}_{x,m}$ from single measurements of $f(0,{150)}_{x,\text{PMMA}}$. Based on comparisons shown in this study and those reported in previous studies,^11^, ^15^, ^16^ the conversion factors only showed a weak dependency on scanner type, thus they may be suitable for application to different CT and CBCT scanners.

## ACKNOWLEDGMENTS

The authors wish to acknowledge the assistance of the support team of Scottish Grid Service (ScotGrid) at University of Glasgow for their support in running the MC codes in ScotGrid. The first author wishes to thank KACST for the scholarship.

## Appendix A: Conversion factors for evaluating f(0, ∞)_x,m_

Tables A.1 to A.3 contain coefficients for fitted polynomial equations to calculate the conversion factors $C_{f}(\text{PMMA}/{\text{PMMA})}_{x,\text{kV}}$, $C_{f}(\text{PMMA}/{\text{PE})}_{x,\text{kV}}$, and $C_{f}(\text{PMMA}/{\text{Water})}_{x,\text{kV}}$. These allow $f(0,{\infty )}_{x,m}$ within infinitely long PMMA, PE, and water head or body phantoms to be evaluated from measurements made in the standard PMMA head or body phantoms using Eq. (5) for beams of width 40−500 mm at tube potentials of 80−140 kV. The conversion factors were fitted to sixth‐degree polynomial equations with $R^{2}>0.99$ and calculated as:

(A1) $$Cf=P_{1}W^{6}+P_{2}W^{5}+P_{3}W^{4}+P_{4}W^{3}+P_{5}W^{2}+P_{6}W+P_{7}$$

where *W* is the beam width in mm.

**Table A.1:** Coefficients for fitted equations to calculate the conversion factors $C_{f}(\text{PMMA}/{\text{PMMA})}_{x,\text{kV}}$ to covert $f(0,{150)}_{x,\text{PMMA}}$ measurements made within the standard PMMA head and body phantoms to $f(0,{\infty )}_{x,\text{PMMA}}$ within infinitely long PMMA head and body phantoms, as in Eq. (5). The conversion factors are suitable for beams of width 40−500 mm at tube potentials of 80−140 kV

	*80 kV*		*100 kV*		*120 kV*		*140 kV*	
*Coefficients*	*Center*	*Periphery*	*Center*	*Periphery*	*Center*	*Periphery*	*Center*	*Periphery*
*Head* – $C_{f}(\text{PMMA}/{\text{PMMA})}_{x,\text{kV}}$								
$P_{1}$	$-7.785\times 10^{-16}$	$-3.793\times 10^{-16}$	$-9.064\times 10^{-16}$	$-4.597\times 10^{-16}$	$-9.411\times 10^{-16}$	$-3.897\times 10^{-16}$	$-8.206\times 10^{-16}$	$-4.657\times 10^{-16}$
$P_{2}$	$1.248\times 10^{-12}$	$6.029\times 10^{-13}$	$1.437\times 10^{-12}$	$7.181\times 10^{-13}$	$1.485\times 10^{-12}$	$6.250\times 10^{-13}$	$1.312\times 10^{-12}$	$7.352\times 10^{-13}$
$P_{3}$	$-7.426\times 10^{-10}$	$-3.560\times 10^{-10}$	$-8.476\times 10^{-10}$	$-4.170\times 10^{-10}$	$-8.715\times 10^{-10}$	$-3.722\times 10^{-10}$	$-7.818\times 10^{-10}$	$-4.314\times 10^{-10}$
$P_{4}$	$1.965\times 10^{-07}$	$9.365\times 10^{-08}$	$2.230\times 10^{-07}$	$1.081\times 10^{-07}$	$2.281\times 10^{-07}$	$9.848\times 10^{-08}$	$2.076\times 10^{-07}$	$1.129\times 10^{-07}$
$P_{5}$	$-2.134\times 10^{-05}$	$-1.012\times 10^{-05}$	$-2.422\times 10^{-05}$	$-1.156\times 10^{-05}$	$-2.461\times 10^{-05}$	$-1.063\times 10^{-05}$	$-2.265\times 10^{-05}$	$-1.222\times 10^{-05}$
$P_{6}$	$1.002\times 10^{-03}$	$4.564\times 10^{-04}$	$1.134\times 10^{-03}$	$5.245\times 10^{-04}$	$1.144\times 10^{-03}$	$4.913\times 10^{-04}$	$1.088\times 10^{-03}$	$5.605\times 10^{-04}$
$P_{7}$	0.9899	0.9957	0.9901	0.9948	0.9910	0.9954	0.9910	0.9946
*Body* – $C_{f}(\text{PMMA}/{\text{PMMA})}_{x,\text{kV}}$								
$P_{1}$	$-1.298\times 10^{-15}$	$-2.901\times 10^{-16}$	$-1.587\times 10^{-15}$	$-3.892\times 10^{-16}$	$-1.498\times 10^{-15}$	$-3.560\times 10^{-16}$	$-1.522\times 10^{-15}$	$-3.574\times 10^{-16}$
$P_{2}$	$2.126\times 10^{-12}$	$4.664\times 10^{-13}$	$2.581\times 10^{-12}$	$6.359\times 10^{-13}$	$2.457\times 10^{-12}$	$5.837\times 10^{-13}$	$2.477\times 10^{-12}$	$5.861\times 10^{-13}$
$P_{3}$	$-1.304\times 10^{-09}$	$-2.808\times 10^{-10}$	$-1.579\times 10^{-09}$	$-3.896\times 10^{-10}$	$-1.512\times 10^{-09}$	$-3.595\times 10^{-10}$	$-1.516\times 10^{-09}$	$-3.602\times 10^{-10}$
$P_{4}$	$3.608\times 10^{-07}$	$7.597\times 10^{-08}$	$4.391\times 10^{-07}$	$1.080\times 10^{-07}$	$4.219\times 10^{-07}$	$1.001\times 10^{-07}$	$4.216\times 10^{-07}$	$9.983\times 10^{-08}$
$P_{5}$	$-4.197\times 10^{-05}$	$-8.511\times 10^{-06}$	$-5.263\times 10^{-05}$	$-1.276\times 10^{-05}$	$-5.037\times 10^{-05}$	$-1.181\times 10^{-05}$	$-5.031\times 10^{-05}$	$-1.164\times 10^{-05}$
$P_{6}$	$2.153\times 10^{-03}$	$4.160\times 10^{-04}$	$2.827\times 10^{-03}$	$6.592\times 10^{-04}$	$2.719\times 10^{-03}$	$6.321\times 10^{-04}$	$2.734\times 10^{-03}$	$6.172\times 10^{-04}$
$P_{7}$	0.9871	0.9964	0.9785	0.9933	0.9813	0.9933	0.9805	0.9939

**Table A.2:** Coefficients of fitted equations to calculate the conversion factors $C_{f}(\text{PMMA}/{\text{PE})}_{x,\text{kV}}$ to covert $f(0,{150)}_{x,\text{PMMA}}$ measurements made within the standard PMMA head and body phantoms to $f(0,{\infty )}_{x,\text{PE}}$ within infinitely long PE head and body phantoms, as in Eq. (5). The conversion factors are suitable for beams of width 40−500 mm at tube potentials of 80−140 kV

	*80 kV*		*100 kV*		*120 kV*		*140 kV*	
*Coefficients*	*Center*	*Periphery*	*Center*	*Periphery*	*Center*	*Periphery*	*Center*	*Periphery*
*Head* – $C_{f}(\text{PMMA}/{\text{PE})}_{x,\text{kV}}$								
$P_{1}$	$-4.650\times 10^{-16}$	$-4.884\times 10^{-16}$	$-4.307\times 10^{-16}$	$-5.701\times 10^{-16}$	$-5.628\times 10^{-16}$	$-5.002\times 10^{-16}$	$-3.823\times 10^{-16}$	$-5.040\times 10^{-16}$
$P_{2}$	$6.672\times 10^{-13}$	$8.054\times 10^{-13}$	$6.231\times 10^{-13}$	$9.317\times 10^{-13}$	$8.005\times 10^{-13}$	$8.263\times 10^{-13}$	$5.370\times 10^{-13}$	$8.280\times 10^{-13}$
$P_{3}$	$-3.149\times 10^{-10}$	$-5.106\times 10^{-10}$	$-2.906\times 10^{-10}$	$-5.826\times 10^{-10}$	$-3.802\times 10^{-10}$	$-5.218\times 10^{-10}$	$-2.346\times 10^{-10}$	$-5.190\times 10^{-10}$
$P_{4}$	$3.686\times 10^{-08}$	$1.568\times 10^{-07}$	$2.798\times 10^{-08}$	$1.746\times 10^{-07}$	$4.902\times 10^{-08}$	$1.573\times 10^{-07}$	$1.073\times 10^{-08}$	$1.547\times 10^{-07}$
$P_{5}$	$1.022\times 10^{-05}$	$-2.534\times 10^{-05}$	$1.267\times 10^{-05}$	$-2.677\times 10^{-05}$	$1.053\times 10^{-05}$	$-2.407\times 10^{-05}$	$1.559\times 10^{-05}$	$-2.325\times 10^{-05}$
$P_{6}$	$-2.008\times 10^{-03}$	$2.695\times 10^{-03}$	$-2.398\times 10^{-03}$	$2.614\times 10^{-03}$	$-2.386\times 10^{-03}$	$2.366\times 10^{-03}$	$-2.735\times 10^{-03}$	$2.244\times 10^{-03}$
$P_{7}$	1.088	0.8088	1.112	0.8528	1.1290	0.8851	1.1490	0.9077
*Body* – $C_{f}(\text{PMMA}/{\text{PE})}_{x,\text{kV}}$								
$P_{1}$	$-1.202\times 10^{-15}$	$-4.025\times 10^{-16}$	$-1.226\times 10^{-15}$	$-3.559\times 10^{-16}$	$-1.169\times 10^{-15}$	$-5.269\times 10^{-16}$	$-1.449\times 10^{-15}$	$-4.838\times 10^{-16}$
$P_{2}$	$1.924\times 10^{-12}$	$6.739\times 10^{-13}$	$1.968\times 10^{-12}$	$6.242\times 10^{-13}$	$1.863\times 10^{-12}$	$8.876\times 10^{-13}$	$2.298\times 10^{-12}$	$8.094\times 10^{-13}$
$P_{3}$	$-1.125\times 10^{-09}$	$-4.372\times 10^{-10}$	$-1.159\times 10^{-09}$	$-4.202\times 10^{-10}$	$-1.080\times 10^{-09}$	$-5.751\times 10^{-10}$	$-1.344\times 10^{-09}$	$-5.214\times 10^{-10}$
$P_{4}$	$2.766\times 10^{-07}$	$1.385\times 10^{-07}$	$2.890\times 10^{-07}$	$1.361\times 10^{-07}$	$2.592\times 10^{-07}$	$1.790\times 10^{-07}$	$3.367\times 10^{-07}$	$1.616\times 10^{-07}$
$P_{5}$	$-2.099\times 10^{-05}$	$-2.329\times 10^{-05}$	$-2.281\times 10^{-05}$	$-2.280\times 10^{-05}$	$-1.685\times 10^{-05}$	$-2.831\times 10^{-05}$	$-2.813\times 10^{-05}$	$-2.556\times 10^{-05}$
$P_{6}$	$7.761\times 10^{-05}$	$2.643\times 10^{-03}$	$5.196\times 10^{-05}$	$2.556\times 10^{-03}$	$-5.470\times 10^{-04}$	$2.815\times 10^{-03}$	$1.341\times 10^{-04}$	$2.620\times 10^{-03}$
$P_{7}$	1.1460	0.6870	1.139	0.7315	1.1560	0.7617	1.1470	0.7803

**Table A.3:** Coefficients for the fitted equations to calculate the conversion factors $C_{f}(\text{PMMA}/{\text{Water})}_{x,\text{kV}}$ to covert $f(0,{150)}_{x,\text{PMMA}}$ measurements made within standard PMMA head and body phantoms to $f(0,{\infty )}_{x,Water}$ within infinitely long water head and body phantoms, as in Eq. (5). The conversion factors are suitable for beams of width 40−500 mm at tube potentials of 80−140 kV

	*80 kV*		*100 kV*		*120 kV*		*140 kV*	
*Coefficients*	*Center*	*Periphery*	*Center*	*Periphery*	*Center*	*Periphery*	*Center*	*Periphery*
*Head* – $C_{f}(\text{PMMA}/{\text{Water})}_{x,\text{kV}}$								
$P_{1}$	$-5.643\times 10^{-16}$	$-7.835\times 10^{-16}$	$-7.507\times 10^{-16}$	$-8.907\times 10^{-16}$	$-8.374\times 10^{-16}$	$-8.207\times 10^{-16}$	$-7.737\times 10^{-16}$	$-7.727\times 10^{-16}$
$P_{2}$	$8.694\times 10^{-13}$	$1.318\times 10^{-12}$	$1.164\times 10^{-12}$	$1.478\times 10^{-12}$	$1.305\times 10^{-12}$	$1.372\times 10^{-12}$	$1.190\times 10^{-12}$	$1.291\times 10^{-12}$
$P_{3}$	$-4.764\times 10^{-10}$	$-8.562\times 10^{-10}$	$-6.549\times 10^{-10}$	$-9.458\times 10^{-10}$	$-7.432\times 10^{-10}$	$-8.866\times 10^{-10}$	$-6.671\times 10^{-10}$	$-8.328\times 10^{-10}$
$P_{4}$	$1.017\times 10^{-07}$	$2.698\times 10^{-07}$	$1.534\times 10^{-07}$	$2.933\times 10^{-07}$	$1.799\times 10^{-07}$	$2.778\times 10^{-07}$	$1.572\times 10^{-07}$	$2.605\times 10^{-07}$
$P_{5}$	$-3.481\times 10^{-06}$	$-4.324\times 10^{-05}$	$-1.086\times 10^{-05}$	$-4.638\times 10^{-05}$	$-1.484\times 10^{-05}$	$-4.453\times 10^{-05}$	$-1.193\times 10^{-05}$	$-4.184\times 10^{-05}$
$P_{6}$	$-5.984\times 10^{-04}$	$3.795\times 10^{-03}$	$-1.699\times 10^{-05}$	$4.107\times 10^{-03}$	$3.152\times 10^{-04}$	$4.061\times 10^{-03}$	$2.188\times 10^{-04}$	$3.903\times 10^{-03}$
$P_{7}$	1.0320	0.9997	1.025	0.9599	1.0150	0.9362	1.0120	0.9201
*Body* – $C_{f}(\text{PMMA}/{\text{Water})}_{x,\text{kV}}$								
$P_{1}$	$-1.093\times 10^{-15}$	$-5.884\times 10^{-16}$	$-1.380\times 10^{-15}$	$-6.676\times 10^{-16}$	$-1.203\times 10^{-15}$	$-6.76\times 10^{-16}$	$-1.674\times 10^{-15}$	$-7.497\times 10^{-16}$
$P_{2}$	$1.757\times 10^{-12}$	$1.015\times 10^{-12}$	$2.190\times 10^{-12}$	$1.157\times 10^{-12}$	$1.966\times 10^{-12}$	$1.15\times 10^{-12}$	$2.677\times 10^{-12}$	$1.272\times 10^{-12}$
$P_{3}$	$-1.030\times 10^{-09}$	$-6.788\times 10^{-10}$	$-1.278\times 10^{-09}$	$-7.751\times 10^{-10}$	$-1.177\times 10^{-09}$	$-7.65\times 10^{-10}$	$-1.588\times 10^{-09}$	$-8.358\times 10^{-10}$
$P_{4}$	$2.508\times 10^{-07}$	$2.211\times 10^{-07}$	$3.188\times 10^{-07}$	$2.517\times 10^{-07}$	$2.990\times 10^{-07}$	$2.46\times 10^{-07}$	$4.122\times 10^{-07}$	$2.667\times 10^{-07}$
$P_{5}$	$-1.658\times 10^{-05}$	$-3.676\times 10^{-05}$	$-2.576\times 10^{-05}$	$-4.154\times 10^{-05}$	$-2.428\times 10^{-05}$	$-4.07\times 10^{-05}$	$-3.936\times 10^{-05}$	$-4.345\times 10^{-05}$
$P_{6}$	$-1.023\times 10^{-03}$	$3.384\times 10^{-03}$	$-3.284\times 10^{-04}$	$3.823\times 10^{-03}$	$-2.269\times 10^{-04}$	$3.844\times 10^{-03}$	$6.570\times 10^{-04}$	$4.029\times 10^{-03}$
$P_{7}$	1.2700	1.1140	1.265	1.062	1.2580	1.024	1.2410	1.003
